# Increased Incidence of Inflammatory Bowel Disease in Association with Dietary Transition (Westernization) in Japan

**DOI:** 10.31662/jmaj.2021-0038

**Published:** 2021-09-27

**Authors:** Mitsuro Chiba, Norikazu Morita, Akira Nakamura, Keisuke Tsuji, Emiko Harashima

**Affiliations:** 1 Division of Gastroenterology, Akita City Hospital, Akita, Japan; 2 Division of Gastroenterology, Morita GI Clinic, Fukuoka, Japan; 3 Professor Emeritus, Department of Medical Information Science, Akita University School of Medicine, Akita, Japan; 4 Laboratory of Environment for Life and Living, School of Humanities for Environmental Policy and Technology, University of Hyogo, Himeji, Japan; 5 Nutrition and Life Science, Kanagawa Institute of Technology, Atsugi, Japan

**Keywords:** Crohn’s disease, ulcerative colitis, epidemiology, dietary westernization, environmental factor, inflammatory bowel disease, incidence

## Abstract

**Introduction:**

Inflammatory bowel disease has become a global disease, but its key environmental factors still remain unrecognized. This study aimed to clarify the role of dietary transition (westernization) in the increased incidence of inflammatory bowel disease in Japan.

**Methods:**

Annual numbers of new cases of inflammatory bowel disease in Japan over the period from 1965 to 2000 found in a nationwide database compiled by the government and the daily amount of food and nutrient intake per capita for the same period revealed by the National Nutrition Survey have been used to analyze their interrelation.

**Results:**

Rapid increases in the estimated incidence per 100,000 population have been observed, that is, from 0.08 in 1965 to 4.8 in 2000 for ulcerative colitis and from 0.003 to 1.3 in 2000 for Crohn’s disease, with an extremely high correlation between the annual numbers of new cases of the respective diseases (r = 0.970). Intake of both animal fat and animal protein increased, while intake of rice decreased during the period. Of all food groups, the intake of rice as a staple food showed the highest negative correlation coefficient with the numbers of new cases of both ulcerative colitis (r = -0.825, 95% CI: -0.908 to -0.681, *p* < 0.0001) and Crohn’s disease (r = -0.836, 95% CI: -0.914 to -0.700, *p* < 0.0001).

**Conclusions:**

An increased incidence of inflammatory bowel disease was observed to coincide with dietary westernization in Japan. Our results support the assertion that dietary westernization is a key environmental factor in inflammatory bowel disease.

## Introduction

Although ulcerative colitis (UC) and Crohn’s disease (CD) form distinct disease entities, they share common features and are collectively referred to as inflammatory bowel disease (IBD). The incidence of IBD in the western world (the USA, Canada, Western Europe, Australia, New Zealand) increased in the latter half of the twentieth century and then plateaued ^(1)^. The prevalence of IBD in these countries exceeds 0.3% of the population. On the other hand, the incidence of IBD has been rapidly increasing since the turn of the twenty-first century in newly industrialized countries in Asia, South America, Eastern Europe, and Africa ^(2)^. Consequently, IBD is recognized as a global disease ^(1), (2), (3)^. Although Japan has the highest prevalence in Asia (UC 219,685, CD 70,700, total population 127.1 million in 2015: prevalence per 100,000 population 172.9 for UC and 55.6 for CD) ^(4)^, it is 0.2% of the population. This is less than that of the western world.

The etiology of IBD is unknown, but it is believed to be multifactorial, whereby development of the disease in genetically susceptible subjects is triggered by environmental factors ^(3)^. Since IBD is prevalent in developed countries, risk factors in affluent societies have been sought. Formula feeding, oral contraceptives, antibiotics in childhood, air pollution, smoking, appendectomy, improved sanitation, decreased physical activity, increased obesity prevalence, and increased stress level, among others, have been listed as risk factors for IBD. However, none of those has been identified as a key environmental factor. Identification and recognition of key environmental factors are prerequisite for effective treatment and prevention of the disease. It has been anticipated that key environmental factors can be identified through meticulous analysis of environmental factors in areas where the incidence of IBD is growing ^(5)^. However, noticeable environmental factors have not been reported in the abovementioned new areas, where the incidence of IBD has rapidly increased since the turn of the twenty-first century ^(1), (2), (3), (6)^.

Recent studies in basic medicine have shed light on the interplay between diet, gut microbiota, microbial metabolites, and health/disease ^(7), (8), (9), (10)^. They have shown that a gut microbial imbalance (dysbiosis) exists in a variety chronic diseases, including obesity, diabetes mellitus, coronary heart disease, and so on, and that our meals shape gut microbiota. Thus, the role of diet is emphasized more than ever in various common diseases. Gut microbial dysbiosis is consistently observed in IBD ^(11)^, implying that improper diet may be involved in onset of IBD.

There have been many ecologic studies, case-control studies, and cohort studies on the relation between diet and IBD ^(12), (13), (14), (15), (16), (17)^. They looked at individual nutrients or foods. Some constituents of diets were certainly found to be risk/preventive factor, but results were often inconsistent. Foods influence each other in a complex manner. Therefore, the overall effect of dietary pattern could be more predictive of disease risk ^(18)^. The results of dietary pattern were again inconsistent. A Mediterranean diet was associated with a lower risk of later onset of CD but not with UC in a Swedish cohort ^(19)^, while no association was found with risk of CD or UC in the European Prospective Investigation into Cancer and Nutrition (EPIC) cohort ^(20)^.

Socioeconomic transition toward an affluent society inevitably induces dietary transition ^(21), (22)^. In the contemporary era, it is dietary westernization characterized by increased consumption of animal fat, animal protein, and sugar with decreased consumption of carbohydrates ^(21), (22)^. For evaluating dietary factors in IBD, it might be more adequate to look at dietary transition as a whole. However, there have been no studies on the relation between dietary transition and the incidence of IBD ^(14), (15), (16), (17), (18), (19)^. Japan provides excellent conditions for such a study for several reasons. First, there is a record of nationwide systemic research on the epidemiology of IBD starting in 1965. Second, a national record of IBD registration has been available since 1973. Third, there is a chronological national record of food intake kept by the National Health and Nutrition Survey. Fourth, the Japanese population is rather homogeneous in terms of genes and culture. Fifth, Japan has a high standard of public health and medicine, as shown by its long average life expectancy ^(23)^. Sixth, the rate of familial aggregation of IBD is low compared with other countries ^(24), (25)^.

Using the above data from between 1966 and 1985 in Japan, Shoda et al. ^(26)^ reported that increased intake of animal protein contributed to the increased incidence of CD, but they did not investigate UC.

We hypothesized that there will be a correlation between dietary westernization and an increased incidence of IBD. In order to clarify the role of dietary factors in the increased incidence of IBD, using the earliest Japanese epidemiological data from 1965 to 2000, we investigated the correlation between dietary transition and the number of new cases of IBD and the correlation between CD and UC in the annual numbers of new cases. A part of this study was previously reported ^(27)^.

## Materials and Methods

### Study settings

The nationwide epidemiological survey of IBD started in 1965 in Japan. The investigation of the correlation between dietary change and the number of new cases of IBD was designed using the national record of IBD and food intake from 1965 to 2000 in Japan. The investigation of the correlation between CD and UC in the annual numbers of cases was also designed using the national registration record from 1977 to 2000.

### Epidemiological data on inflammatory bowel disease

The Ministry of Health, Labor and Welfare (formerly the Ministry of Health and Welfare) of Japan designated UC and CD as intractable diseases in 1975 and 1976, respectively. Patients with intractable diseases are provided with public medical aid upon registration at a public health office. Patients diagnosed with IBD are required to submit a registration application form annually to the local government ^(28)^. The attending doctor provides detailed information about the patient’s condition on the form. The submitted forms are evaluated by IBD specialists on each prefecture’s committee for IBD. Those who are definitively diagnosed become eligible for registration and for financial medical aid ^(28)^. The annual number of registrations is available ^(29), (30), (31)^. The number of IBD registrations is estimated to account for about 80% of patients with IBD since some patients with IBD do not apply for the aid because they either do not need it or are not willing to be registered as having an intractable disease ^(32)^. The numbers of new cases were arbitrarily defined as the increase in registrations over the preceding year. The annual numbers of new cases since 1965 to the beginning of the national registration were obtained from nationwide hospital-based epidemiological studies conducted by the Research Committee for Intractable Intestinal Diseases, Ministry of Health and Welfare ^(33), (34)^.

### Nutritional data

Data on dietary intake per capita per day were obtained from the National Nutrition Survey reports, except for dietary fiber intake ^(35)^. The Ministry of Health, Labor and Welfare has been conducting the National Nutrition Survey every year since 1946. Briefly, approximately 15,000 people from 5,000 families are selected at random among 300 area units nationwide. Staff dietitians from the local public health center visit respondent households for 3 consecutive days, provide guidance on how to fill in the questionnaire, check the records, and correct any inadequacies. The quantity of food intake is calculated from the recorded dietary intake, and then the quantity of nutrient intake is calculated from the quantity of food intake based on food composition tables ^(35)^. The National Nutrition Survey covers 15 food groups and several nutrients ^(35)^. Data on dietary fiber intake per capita were obtained from the intake of grain, vegetables and mushrooms, beans, fruits, potatoes, seaweed, and confectionaries as described in a previous report by two of the authors (K.T., E.H.) and colleagues ^(36)^.

### Statistical analysis

The interrelationships between the annual numbers of new cases and cases of UC and CD from 1977 to 2000 were determined using multiple Pearson product-moment correlation coefficients and were fitted to a linear approximation. Interrelationships between food/nutrient intakes and the numbers of new IBD cases from 1965 to 2000 were determined using multiple Pearson product-moment correlation coefficients. Multiple correlation and multiple regression analyses were done between food intakes and the numbers of new IBD cases. Factor analysis was performed among six nutrients and energy and the numbers of new IBD cases, by extraction of the principal component followed by the Varimax rotation. All tests were two-tailed; a *P* value of 0.05 or less was considered to indicate a statistically significant difference. Statistical analyses were performed using SPSS for Windows v10.0.7J (SPSS Japan Inc. Tokyo, Japan) on Windows 2000 OS using a Sony VAIO computer (Sony Corp., Tokyo, Japan) and StatView v4.5 (Abacus Concepts Inc., Berkeley, CA, USA) using a Macintosh PowerBook G3 Series computer (Apple Computer, Inc., Cupertino, CA, USA).

## Results

### Epidemiological data on IBD

**Figure 1.**
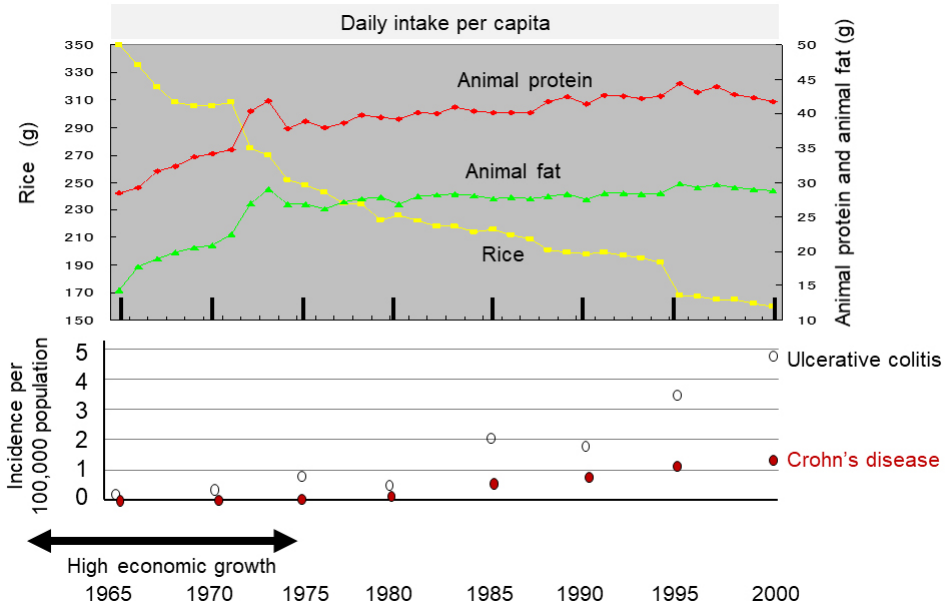
Chronological change in dietary intake and incidence of IBD in Japan. The daily intake per capita of rice, animal protein, and animal fat is shown in the upper panel based on the data for 35 years from 1965 to 2000 obtained by the National Nutritional Survey ^(35)^. The incidence of IBD every 5 years (Table 1) is shown in the lower panel.

Table 1 summarizes the number of IBD patients every 5 years between 1965 and 2000. There were 79 new cases of UC and 3 cases of CD in 1965, while there were 6083 new cases of UC and 1615 cases of CD in 2000 (Table 1). The estimated incidence of IBD per 100,000 population in 1965 and 2000 was 0.08 and 4.8, respectively, for UC, and 0.003 and 1.3, respectively, for CD (Table 1 and Figure 1). They represent a 60-fold and 433-fold increase in the estimated incidence of UC and CD, respectively, after 35 years. In 2000, there were 66,714 patients with UC and 19,651 patients with CD (Table 1).

**Table 1.** Number of Patients with Inflammatory Bowel Disease and Dietary Intake in Japan.

	1965	1970	1975	1980	1985	1990	1995	2000
Number of new cases								
Ulcerative colitis	79	256	965	432	2490	2387	4264	6083
Crohn’s disease	3	12	82	122	656	894	1308	1615
Number of patients								
Ulcerative colitis			965	4405	11602	23200	41243	66714
Crohn’s disease			128*	672	2831	6694	12645	19651
	
Total population by census (million)	98.3	103.7	111.9	117.1	121.0	123.6	125.6	127.0
Estimated incidence per 100,000								
Ulcerative colitis	0.08	0.25	0.9	0.4	2.1	1.9	3.4	4.8
Crohn’s disease	0.003	0.01	0.07	0.1	0.5	0.7	1.0	1.3
	
Food groups intake per capita/day								
Rice (g)	350	306	248	226	216	198	168	160
Wheat (g)	60.4	64.8	90.2	91.8	91.3	84.8	93.7	94.3
Potatoes (g)	41.9	37.8	60.9	63.4	63.2	65.3	68.9	64.7
Sugars (g)	17.9	19.7	14.6	12.0	11.2	10.6	9.9	9.3
Confectioneries (g)	31.6	36.7	29.0	25.0	22.8	20.3	26.8	22.2
Fats & oils (g)	10.2	15.6	15.8	16.9	17.7	17.6	17.3	16.4
Beans (g)	69.6	71.2	70.0	65.4	66.6	68.5	70.0	70.2
Fruits (g)	58.8	81.0	193.5	155.2	140.6	124.8	133.0	117.4
Vegetables & mushrooms (g)	219.4	249.3	246.7	251.4	261.7	250.3	290.2	276.0
Seaweeds (g)	6.1	6.9	4.9	5.1	5.6	6.1	5.3	5.5
Beverages (g)	119.4	163.4	148.4	134.7	136.1	137.4	190.2	182.3
Fishes & shellfishes (g)	76.3	87.4	94.0	92.5	90.0	95.3	96.9	92.0
Meats (g)	29.5	42.5	64.2	67.9	71.7	71.2	82.3	78.2
Eggs (g)	35.2	41.2	41.5	37.7	40.3	42.3	42.1	39.7
Milk & dairy products (g)	57.4	78.8	103.5	115.2	116.7	130.1	144.4	127.6
Energy (kcal)	2184	2210	2188	2084	2088	2026	2042	1948
Nutrient intake per capita/day								
Carbohydrate (g)	384	368	337	313	298	287	280	266
Protein (g)	71.3	77.6	81.0	78.7	79.0	78.7	81.5	77.7
Fat (g)	36.0	46.5	52.0	52.4	56.9	56.9	59.9	57.4
Dietary fiber (g)	15.7	16.3	16.5	15.7	15.8	15.2	15.9	15.7
Animal protein (g)	28.5	34.2	38.9	39.2	40.1	41.0	43.9	41.7
Animal fat (g)	14.3	20.9	26.9	26.9	27.6	27.5	29.8	28.8

*The registration of Crohn’s disease started in 1976. The figure in 1976 is presented.

**Figure 2.**
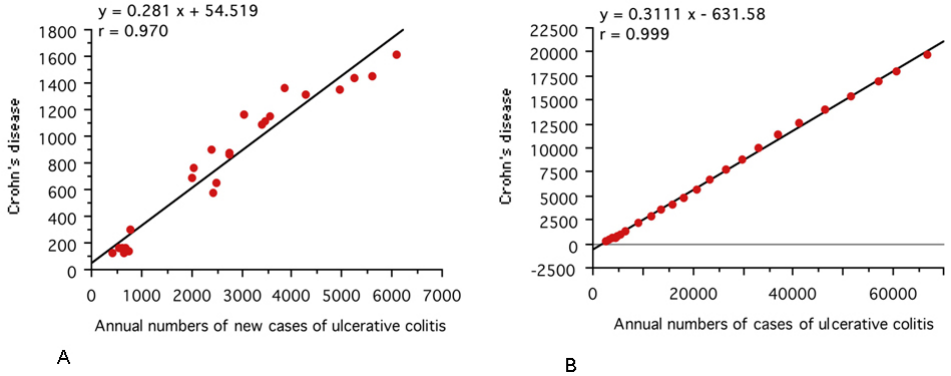
Correlation between Crohn’s disease and ulcerative colitis in the annual numbers of new cases (A) and the annual numbers of cases (B). In Japan, national registration of ulcerative colitis and Crohn’s disease started in 1975 and 1976, respectively. A scattergram was generated based on the data for 24 years from 1977 to 2000 ^(29), (30), (31)^. The linear regression formula and correlation coefficient are shown.

An extremely high correlation was observed between UC and CD in the annual numbers of new cases (r = 0.970, 95% CI: 0.931-0.987,* p* <0.0001) and the annual numbers of cases (r = 0.999, 95% CI: 0.999-1.000,* p* <0.0001) (Figure 2).

### Dietary intake

#### Chronologic change in dietary intake

Table 1 summarizes the daily dietary intake for all 15 food groups, 6 nutrients (carbohydrates, protein, fat, dietary fiber, animal protein, and animal fat), and energy every 5 years between 1965 and 2000. Intake of total energy, sugars, and confectioneries peaked around 1970 and then started to decrease (Table 1). Intake of both animal fat and animal protein increased, while intake of rice decreased. These changes occurred drastically from 1965 to 1973 (Figure 1). Thereafter, increased intake of animal fat and animal protein was slight, but a decrease in rice intake occurred at a similar pace for 35 years (Table 1 and Figure 1).

#### Correlation of dietary intake among food groups and nutrients

Correlations of different strengths can be identified among the 15 food groups, energy, and 6 nutrients (Table 2). Rice intake decreased year by year and was strongly negatively correlated to intake of milk and dairy products (r = -0.974, 95% CI: -0.987 to -0.949, *p* < 0.0001), meats (r = -0.926, 95% CI: -0.962 to -0.85, *p* < 0.0001), animal protein (r = -0.924, 95% CI: -0.961 to -0.854, *p* < 0.0001), and animal fat (r = -0.897, 95% CI: -0.947 to -0.806, *p* < 0.0001) (Table 2).

**Table 2.** Simple Correlation Coefficients and Their P Values among Nutritional Variables and Inflammatory Bowel Disease.

	Ri	Wh	Po	Su	Co	FO	Be	Fr	VM	SW	Bev	FS	Me	Eg	MD	En	CH	P	F	DF	AP	AF	UC	CD
Ri	1.000	-0.795	-0.805	0.895	0.761	-0.722	0.267	-0.250	-0.578	0.164	-0.406	-0.833	-0.926	-0.540	-0.974	0.880	0.985	-0.522	-0.927	0.525	-0.924	-0.897	-0.825	-0.836
Wh	****	1.000	0.759	-0.767	-0.630	0.652	-0.546	0.723	0.584	-0.554	0.127	0.693	0.896	0.319	0.781	-0.522	-0.764	0.683	0.871	-0.129	0.819	0.904	0.426	0.405
Po	****	****	1.000	-0.855	-0.846	0.483	-0.135	0.392	0.372	-0.497	-0.034	0.687	0.759	0.206	0.737	-0.756	-0.822	0.364	0.737	-0.363	0.693	0.714	0.618	0.631
Su	****	****	****	1.000	0.866	-0.518	0.402	-0.267	-0.436	0.391	-0.124	-0.808	-0.893	-0.359	-0.870	0.825	0.923	-0.476	-0.805	0.540	-0.866	-0.824	-0.753	-0.787
Co	****	****	****	****	1.000	-0.534	0.281	-0.169	-0.118	0.281	0.186	-0.592	-0.673	-0.266	-0.730	0.830	0.840	-0.156	-0.684	0.681	-0.624	-0.616	-0.602	-0.670
FO	****	****	**	**	***	1.000	-0.381	0.387	0.421	0.055	0.190	0.613	0.719	0.742	0.787	-0.490	-0.698	0.528	0.881	-0.454	0.727	0.771	0.365	0.408
Be		***		*		*	1.000	-0.547	-0.148	0.394	0.217	-0.327	-0.505	-0.234	-0.339	0.034	0.279	-0.472	-0.423	0.680	-0.461	-0.510	0.045	0.012
Fr		****	*			*	***	1.000	0.430	-0.684	-0.085	0.360	0.506	0.169	0.263	0.123	-0.180	0.677	0.487	0.369	0.415	0.580	-0.173	-0.215
VM	***	***	*	**		**		**	1.000	-0.309	0.605	0.619	0.658	0.391	0.542	-0.270	-0.468	0.739	0.602	0.184	0.667	0.711	0.473	0.407
SW		***	**	*			*	****		1.000	0.239	-0.244	-0.362	0.302	-0.104	0.009	0.157	-0.345	-0.204	-0.268	-0.239	-0.348	0.036	0.066
Be									****		1.000	0.333	0.329	0.350	0.383	-0.273	-0.296	0.335	0.304	0.060	0.400	0.334	0.549	0.468
FS	****	****	****	****	****	****		*	****		*	1.000	0.875	0.648	0.843	-0.606	-0.789	0.777	0.838	-0.330	0.929	0.882	0.651	0.677
Me	****	****	****	****	****	****	*	**	****	*	*	****	1.000	0.567	0.926	-0.677	-0.892	0.745	0.948	-0.351	0.981	0.982	0.658	0.666
Eg	***			*		****			*		*	****	***	1.000	0.612	-0.322	-0.482	0.589	0.656	-0.371	0.664	0.621	0.386	0.436
MD	****	****	****	****	****	****	*		***		*	****	****	****	1.000	-0.812	-0.958	0.579	0.948	-0.552	0.942	0.907	0.754	0.787
En	****	***	****	****	****	**						****	****		****	1.000	0.925	-0.091	-0.679	0.704	-0.670	-0.598	-0.877	-0.903
CH	****	****	****	****	****	****			**			****	****	**	****	****	1.000	-0.418	-0.896	0.618	-0.881	-0.847	-0.827	-0.852
P	***	****	*	**		***	**	****	****	*	*	****	****	***	***		*	1.000	0.698	0.171	0.776	0.807	0.233	0.219
F	****	****	****	****	****	****	**	**	****			****	****	****	****	****	****	****	1.000	-0.407	0.939	0.961	0.609	0.625
DF	***		*	***	****	**		*				*	*	*	***	****	****		*	1.000	-0.382	-0.283	-0.543	-0.642
AP	****	****	****	****	****	****	**	*	****		*	****	****	****	****	****	****	****	****	*	1.000	0.971	0.694	0.711
AF	****	****	****	****	****	****	*	***	****	*	*	****	****	****	****	***	****	****	****		****	1.000	0.591	0.593
UC	****	*	****	****	***	*			**		***	****	****	*	****	****	****		****	***	****	****	1.000	0.979
CD	****	*	****	****	****	*			*		**	****	****	**	****	****	****		****	****	****	****	****	1.000

*P < 0.05, **P < 0.01, ***P < 0.001, ****P < 0.0001 Ri, Rice; Wh, wheat; Po, potatoes; Su, sugars; Co, confectioneries; FO, fats & oils; Be, beans; Fr, fruits; VM, vegetables & mushrooms; SW, seaweeds; Bev, beverages; FS, fishes & shellfishes; Me, meets; Eg, eggs; MD, milk & dairy products; En, energy; CH, carbohydrate; P, protein; F, fat; DF, dietary fiber; AP, animal protein; AF, animal fat; UC, ulcerative colitis; CD, Crohn’s disease

### Correlation between new cases of IBD and dietary intake

#### Correlation between new cases of IBD and dietary intake by food group

**Figure 3.**
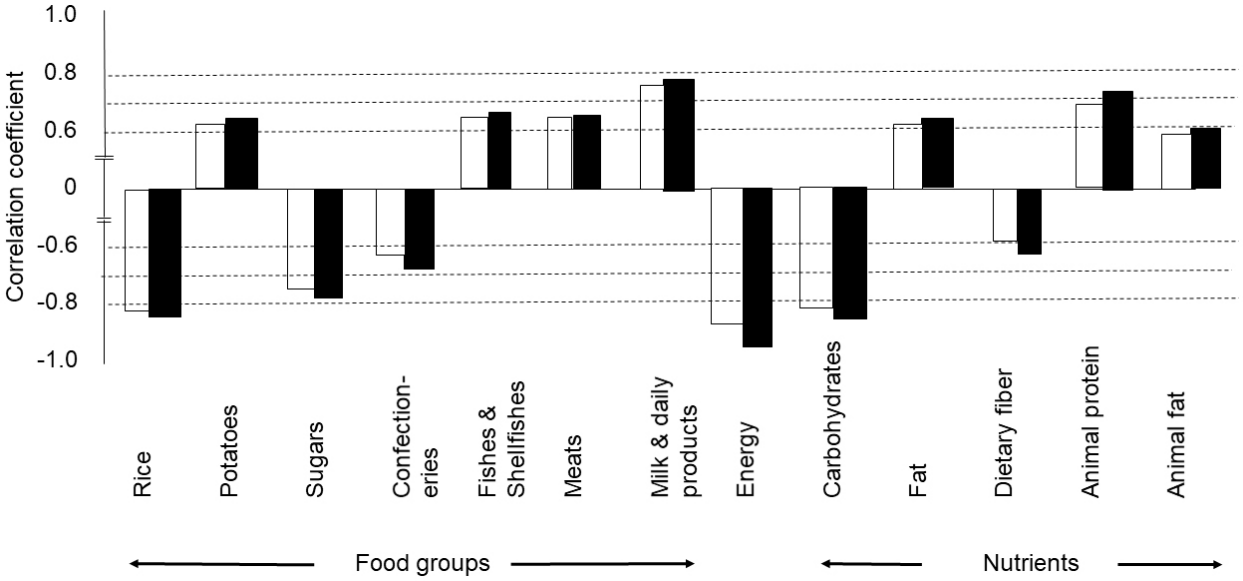
Correlation coefficient between increase in new cases of inflammatory bowel disease and food groups, nutrients, or energy. Data from 1965 to 2000 were used. Foods, nutrients, or energy with a *p* value less than 0.0001 are shown in this figure, except for dietary fiber for ulcerative colitis (*p* < 0.001) (Table 2). The white column shows ulcerative colitis, and the black column shows Crohn’s disease.

UC and CD showed a positive correlation with increased intake of potatoes, fish and shellfish, meats, and milk and dairy products and a negative correlation with intake of rice, sugars, and confectioneries (Table 2 and Figure 3).

Rice intake showed the highest negative correlation coefficient among the 15 food groups to both UC (r = -0.825, 95% CI: -0.908 to -0.681, *p* < 0.0001) and CD (r = -0.836, 95% CI: -0.914 to -0.700, *p* <0.0001) (Table 2 and Figure 3).

For multiple regression analyses, rice, which showed the highest negative correlation coefficient to both UC and CD, was chosen as an explanatory variable. For the explanatory variables, foods with a high correlation coefficient (more than 0.8) to rice or foods with a nonsignificant correlation to UC/CD were excluded. Based on multiple regression analyses, three variables were extracted. Rice had the highest standard regression coefficient among three variables (rice, wheat, and fats and oils) for both UC (-1.670) and CD (-1.678) according to multiple regression analyses (Table 3). The regression coefficients were 0.958 and 0.967 for UC and CD, respectively (Table 3).

**Table 3.** Multiple Linear Regression between Food Intake and the Number of New Cases of Inflammatory Bowel Disease.

Food	Ulcerative colitis				Crohn’s disease			
		Regression coefficient	Standard regression coefficient	*p* value		Regression coefficient	Standard Regression coefficient	*p* value
Rice		-54.463	-1.670	< 0.0001		-17.186	-1.678	< 0.0001
Wheat		-96.753	-0.635	< 0.0001		-34.811	-0.728	< 0.0001
Fats & oils		-354.432	-0.452	< 0.0001		-88.677	-0.360	< 0.0001
	Intercept	28703.128		< 0.0001	Intercept	8894.132		< 0.0001
	R	0.958			R	0.967		

#### Correlation between new cases of IBD and dietary intake of nutrients and energy

UC and CD showed a positive correlation with increased intake of fat, animal protein, and animal fat and a negative correlation with intake of energy, carbohydrates, and dietary fiber (Table 2 and Figure 3).

**Figure 4.**
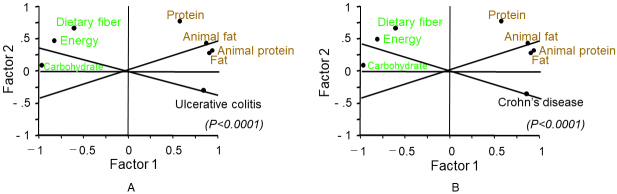
Factor analysis of inflammatory bowel disease (IBD) and intake of nutrients and energy. Trends of variables are similar in both ulcerative colitis (A) and Crohn’s disease (B) (Table 4). Fat, animal protein, and animal fat were identified as promoting factors for IBD; in contrast, carbohydrates, dietary fiber, and energy were identified as prophylactic factors against IBD.

Factor analysis among the six nutrients and energy showed similar results for UC and CD. Factor 1 that comprised of high regression coefficients of animal protein, animal fat, and fat was considered as a westernized diet, and factor 2 that comprised of a high regression coefficient of dietary fiber was regarded as a traditional diet (Table 4). Fat, animal protein, and animal fat in the graph are close to UC/CD, and these were identified as promoting factors for IBD (Table 4 and Figure 4). In contrast, energy, carbohydrates, and dietary fiber were opposite to UC/CD, and these were identified as prophylactic factors against IBD (Figure 4).

**Table 4.** Factor Analysis among Six Nutrients and Energy and the Number of New Cases of Inflammatory Bowel Disease: Ulcerative Colitis (Upper Panel) and Crohn’s Disease (Lower Panel).

Principal component	Eigen value	Proportion of variance	Loading score							
			Energy	Carbo- hydrates	Protein	Fat	Dietary fiber	Animal protein	Animal fat	Ulcerative colitis
Factor 1	5.705	71.3%	-0.842	-0.975	0.637	0.946	-0.550	0.960	0.922	0.819
Factor 2	1.681	21.0%	0.490	0.160	0.747	0.200	0.727	0.245	0.357	-0.318
Factor 3	0.368	4.6%								
Factor 4	0.180	2.3%								
	
**Principal** **component**	**Eigen** **value**	**Proportion** **of** **variance**	**Energy**	**Carbo-** **hydrates**	**Protein**	**Fat**	**Dietary** **fiber**	**Animal** **protein**	**Animal** **fat**	**Crohn's** **disease**
Factor 1	5.729	71.6%	-0.847	-0.976	0.628	0.944	-0.567	0.957	0.917	0.834
Factor 2	1.726	21.6%	0.474	0.143	0.753	0.215	0.725	0.257	0.371	-0.372
Factor 3	0.290	3.6%								
Factor 4	0.196	2.4%								

Overall significance of p < 0.0001 in both upper and lower panels which contain ulcerative colitis and Crohn’s disease, respectively

## Discussion

This study showed that the incidence of IBD was associated with dietary westernization (Table 1 and Figure 1). The characteristics of dietary westernization, i.e., increased intake of animal protein and animal fat with decreased intake of rice, were observed over the survey time. The incidence of IBD rapidly increased during the same period. Analyzing not only selected foods but all food groups and nutrients, this study revealed that the correlation coefficient of decreased rice intake to both UC and CD was higher than those of increased intake of meat, fish and shellfish, and milk and dairy products (Table 2 and Figure 3).

The association of IBD increase and dietary westernization was not specially stated, but it was historically indicated. In the United States, the ratio of fat and carbohydrates to total energy intake gradually increased and decreased, respectively, during the 70 years between 1910 and 1980, i.e., from 33% to 43% and from 55% to 46%, respectively. The protein ratio increased only 1%, from 12% to 13% ^(37)^. This seems to be a gradual dietary westernization. In other western countries, a dramatic dietary transition to a westernized diet occurred after World War II (1941-1945) ^(38)^. A rising incidence of IBD in the west commenced in the 1950s ^(39)^. Therefore, an increase in the incidence of IBD occurred after dietary westernization in the western world. After World War II, Japan experienced an economic reconstruction period (1950-1960) followed by a period of high economic growth (1960-1975) ^(35)^. The decrease in rice intake commenced in 1965 with an increase in intake of animal fat and animal protein (Figure 1) ^(35), (40)^. Dietary transition similar to that in the United States was observed in Japan, although the ratios of macronutrients to total energy intake were different. That is, the ratio of fat intake increased from 14.8% in 1965 to 26.5% in 2000 while that of carbohydrates decreased from 72.1% to 57.5% in the corresponding year, respectively. The ratio of protein intake increased only 2.6%, from 13.3% to 15.9% ^(40)^.

Dietary transition in the United States, Europe, and Japan is typically associated with socioeconomic transition. However, the development of a food industry that produces foods rich in fats at low cost and urbanization induced similar dietary westernization in lower-income countries. Popkin pointed out a global dietary transition from a traditional diet to a westernized diet in Asia, South America, and Africa between 1962 and 1994. They also warned that such a transition was destined to result in an increase in the incidence of chronic diseases, including coronary heart disease, stroke, and diabetes mellitus ^(22), (41)^. The fear became real. The incidence of diet-related obesity and chronic diseases has been steadily increasing, becoming a global health concern ^(42)^. The epidemiology of diet-related chronic diseases and IBD is more or less similar.

As observed above, there might be a great variety in dietary transition (westernization) in terms of speed, degree, and mode of adaption to the transition among countries, races, and individuals based on their culture and tradition. Likewise, other lifestyles associated with socioeconomic transition and related to IBD are heterogenous. This seems to be one of the explanations for inconsistent results or contradictory findings of the relation between diet and IBD ^(12), (13), (14), (15), (16), (17)^. Therefore, cautious comprehensive analysis is needed for interpretation of the results. The lack of consistent evidence has hampered the formulation of dietary guidelines for IBD, with the exception of exclusive enteral nutrition in CD ^(43)^.

Increased sugar consumption is one of the characteristics of dietary westernization, but in the present study, sugar consumption peaked at 20.7 g in 1969 and then started to decrease (Table 1). Consequently, sugar intake was negatively correlated with the increased incidence of IBD (Table 2 and Figure 3). This would be another example of a variety of dietary westernization in which sugar intake was not increased in Japan. Case-control studies in Japan showed that patients with pre-illness CD consumed more sugars than controls ^(44), (45)^.

Extremely high correlations between CD and UC were observed in the annual numbers of new cases and the annual numbers of cases (Figure 2). This is consistent with an earlier report ^(46)^. This observation indicates that there is a common environmental factor in the onset of the two diseases. Cohort studies of dietary fiber, ratio of omega-3/omega-6 polyunsaturated fatty acids, and dietary patterns with high inflammatory potential showed no identical effect for onset of both UC and CD but showed an effect for either UC or CD ^(14), (15), (47)^. Therefore, these factors do not seem to be key factors in IBD. In this study, dietary westernization was associated with the incidence of both UC and CD.

If dietary westernization is associated with onset of IBD, our current (westernized) diet has to be scrutinized. Current global consumption consists of an excess of unhealthy foods such as red meat, sugar, and refined grains and a shortage of healthy foods such as vegetables, fruits, legumes, whole grains, and nuts ^(42), (48)^. The former and the latter largely overlap risk factors and preventive factors, respectively, in IBD ^(12), (13), (14), (15), (16), (17)^. Basic research has revealed that a westernized diet tends to be pro-inflammatory, while a plant-based diet (PBD) tends to be anti-inflammatory ^(9), (10), (11), (12), (38), (49)^. It is a pity that the incidence of IBD has repeatedly increased in regions where IBD was absent or rare. It is easily anticipated that the incidence of chronic diseases and IBD will increase worldwide, unless people, including care providers, notice that the current westernized diet is problematic ^(22), (41), (42), (48)^. The recommended healthy reference diet suggests moderate consumption of animal food and sugar and increased consumption of vegetables and fruits ^(42), (48)^. Considerable moderation of animal foods is categorized as a PBD. PBDs are recommended to the public as a healthy diet to prevent common chronic diseases ^(42), (48)^. We believe that generalization of a healthy reference diet or PBD will decrease the incidence of diet-related chronic diseases, including IBD.

As a matter of fact, the present authors have recognized that IBD is a lifestyle disease mediated mainly by a westernized diet ^(50), (51)^. Therefore, we developed and began to provide a PBD to replace an omnivorous (westernized) diet for Japanese patients with IBD in 2003 ^(50)^. We published far better outcomes in terms of both induction and relapse rate in both diseases as compared with the current standard ^(50), (52), (53), (54)^. Consequently, we recommended PBD for IBD ^(55)^. The present epidemiological study obviously does not show a causal relation between diet and IBD, but it does support our assertion.

Our study has some limitations. The number of new cases between 1965 and 1975 may be underestimated as IBD was rare in those days, and there might be cases undiagnosed as IBD. Food groups differ from country to country. This study did not evaluate fast food, food additives including artificial sweeteners ^(45)^ and emulsifiers ^(56)^, and ultra-processed food ^(57)^, which are associated with the development of the food industry. We hope that other studies will be conducted to validate our results.

In conclusion, the incidence of IBD increased in Japan in association with westernization of the diet. Appreciation of the association of IBD incidence with dietary westernization will change the current therapeutic modality and contribute to establishment of a policy against further increase in the incidence of IBD.

## Article Information

### Conflicts of Interest

None

### Acknowledgement

The authors thank Marcin J. Schroeder, Ph.D., Professor of Mathematics at Akita International University (Present: Specially Appointed Professor, Global Learning Center, Institute for Excellence in Higher Education, Tohoku University), for the statistical review.

### Author Contributions

MC: design, data acquisition, analysis, interpretation, and manuscript writing. NM and AN: data acquisition, analysis, interpretation, and manuscript critical revision. KT and EH: data acquisition and manuscript critical revision. All authors approved the final version of the manuscript for submission.
